# Frequency of genetic variants involved in lipid metabolism and intrahepatic fat

**DOI:** 10.31744/einstein_journal/2024CE1122

**Published:** 2024-09-09

**Authors:** Mariana Cavalheiro Magri, Thamiris Vaz Gago Prata, Bianca Peixoto Dantas, Caroline Manchiero, Gerusa Maria Figueiredo, Fátima Mitiko Tengan

**Affiliations:** 1 Universidade de São Paulo Faculdade de Medicina Hospital das Clínicas São Paulo SP Brazil Laboratório de Investigação Médica em Hepatologia por Vírus (LIM-47), Hospital das Clínicas, Faculdade de Medicina, Universidade de São Paulo, São Paulo, SP, Brazil.; 2 Universidade de São Paulo Instituto de Medicina Tropical de São Paulo Faculdade de Medicina São Paulo SP Brazil Instituto de Medicina Tropical de São Paulo, Faculdade de Medicina, Universidade de São Paulo, São Paulo, SP, Brazil.; 3 Universidade de São Paulo Faculdade de Medicina Department of Preventive Medicine São Paulo SP Brazil Department of Preventive Medicine, Faculdade de Medicina, Universidade de São Paulo, São Paulo, SP, Brazil.; 4 Universidade de São Paulo Faculdade de Medicina Department of Infectious Diseases and Tropical Medicine São Paulo SP Brazil Department of Infectious Diseases and Tropical Medicine, Faculdade de Medicina, Universidade de São Paulo, São Paulo, SP, Brazil.

Dear Editor,

An alarming number of pandemics are affecting the world's population, including viral infections, overweight/obesity, and metabolic disorders. Genetic variants may be involved in underlying mechanisms of lipid metabolism and intrahepatic fat accumulation. The minor allele frequencies (MAFs) of variants in the *microsomal triglyceride transfer protein* (MTTP) gene, which unbalance the concentration of cholesterol, low-density lipoprotein and apolipoprotein B,^(1^^-^^3)^ were investigated in 241 healthy Brazilians.

Data were examined in conjunction with results from the Allele Frequency Aggregator (ALFA) Project (Table 1), in which approximately one million individuals were included.^(4)^ The MAFs of H297Q and -493G/T variants were higher in the Brazilian population, possibly indicating a risk for metabolic disorders. These variations may be attributable to the fact that the ALFA Project was composed of a small Latin American population. Brazil has a unique population with evidence of high levels of genetic admixture,^(5)^ and we also considered epidemiological data on diversity, which is a challenging aspect of precision medicine.

**Table 1:** Minor allele frequencies of genetic variants in the *microsomal triglyceride transfer protein* gene identified in the Brazilian population and in the NCBI SNP database (ALFA Project)

Variant (SNP)	Chr4 position	Allele	MAF				HWE*
			NCBI Global	NCBI Latin American 1	NCBI Latin American 2	Brazilian population	
-164T/C (rs1800804)	99574660	T/C	0.26	0.26	0.17	0.27	0.532
-400A/T (rs1800803)	99574424	A/T	0.37	0.46	0.26	0.37	0.212
H297Q (rs2306985)	99594865	C/G (H/Q)	0.39	0.53	0.27	0.50	0.220
-493G/T (rs1800591)	99574331	G/T	0.15	0.00†	0.00	0.33	0.723
I128T (rs3816873)	99583507	T/C (I/T)	0.26	0.28	0.17	0.26	0.277
Q95H (rs61733139)	99583409	G/C (Q/H)	0.05	0.04	0.02	0.09	0.098
Q244E (rs17599091)	99591762	C/G (Q/E)	0.03	0.04	0.01	0.06	0.302

* p≥0.05 indicates Hardy-Weinberg equilibrium in the Brazilian population (χ^2^ test); † small sample size (n=68).

Chr4: chromosome 4; HWE: Hardy-Weinberg equilibrium; Latin American 1: Latin American individuals with Afro-Caribbean ancestry; Latin American 2: Latin American individuals with mostly European and Native American ancestry; MAF: minor allele frequency; NCBI: National Center for Biotechnology Information; SNP: single nucleotide polymorphism.

The study was approved by the Ethics Committee of the *Hospital das Clinicas,* *Universidade de Sao Paulo* (CAAE: 57626816.3.0000.0068; #2.779.235).
